# The effectiveness of commercial household ultraviolet C germicidal devices in Thailand

**DOI:** 10.1038/s41598-021-03326-4

**Published:** 2021-12-13

**Authors:** Pasita Palakornkitti, Prinpat Pinyowiwat, Somsak Tanrattanakorn, Natta Rajatanavin, Ploysyne Rattanakaemakorn

**Affiliations:** Division of Dermatology, Department of Medicine, Faculty of Medicine Ramathibodi Hospital, Mahidol University, 270 Rama VI Road, Ratchathewi, Bangkok, 10400 Thailand

**Keywords:** Environmental sciences, Health care, Infectious diseases

## Abstract

Ultraviolet C (UVC), or ultraviolet germicidal irradiation (UVGI), is known for its effective air, water, and surface disinfectant properties. With the rise of global awareness about public sanitation and personal hygiene due to the emergence of the current coronavirus disease 2019 pandemic, several applications of UVC were introduced to the commercial market. The present experimental study aimed to evaluate the effectiveness of commercial household UVC germicidal devices for severe acute respiratory syndrome coronavirus 2 (SARS-CoV-2) inactivation. Ten UVC devices were included in the study comprising of 7 low-pressure mercury lamps (LPMLs) and 3 UVC- light-emitting diodes (LEDs). Considering applications, 3 were handheld UVGI surface disinfection equipment, 4 were UVGI disinfection chambers, and 3 were movable UVGI air and surface purifiers. To determine SARS-CoV-2 inactivation performance, UVC irradiance (mW/cm^2^) was measured 3 times repeatedly at distance and duration corresponding to manufacturers’ usage instructions. The required UVC dosage could not be achieved by either of UVC-LED devices (1 handheld UVGI surface disinfection equipment and 2 UVGI disinfection chambers). Five of seven LPMLs can sufficiently emit UVC irradiance for SARS-CoV-2-inactivation. A lack of standardization in the distance and cycle duration for each UVC application was observed. Standard usage guidelines for UVC devices are required to improve the effectiveness of UVC irradiance for SARS-CoV-2 inactivation as well as to minimize the potential side effects of UVC.

## Introduction

The emergence of the current coronavirus disease 2019 (COVID-19) pandemic has raised global awareness about public sanitation and personal hygiene. COVID-19, caused by severe acute respiratory syndrome coronavirus 2 (SARS-CoV-2), was first reported in Wuhan, China, on December 31, 2019, and was identified as a pandemic disease by the World Health Organization on March 11, 2020^1^. The transmission of SARS-CoV-2 primarily occurs through direct contact, indirect contact (fomite transmission), and respiratory droplets^2^. Recent studies have shown potential airborne transmission of SARS-CoV-2. The studies generated aerosols of SARS-CoV-2 from nebulizers under experimental conditions. One study showed that SARS-CoV-2 remains viable within aerosols for 3 hours^3^. Another study suggested that the infectivity and virion integrity of SARS-CoV-2 persisted for up to 16 hours in respirable-sized aerosols^4^.

To control the pandemic, several modalities have been adopted to reduce transmission, including social distancing, use of masks, hand hygiene, disinfectants, and surface cleaning^5^. Among disinfection methods, ultraviolet germicidal irradiation (UVGI) systems are gaining popularity due to their effective disinfectant properties for air, liquids, and surfaces^6–9^.

Ultraviolet radiation is electromagnetic radiation that is classified into UVA (315–400 nm), UVB (280–315 nm), UVC (200–280 nm), and vacuum UV (100–200 nm). UVC is primarily used in UVGI because it has strong germicidal effects, and its wavelength (particularly 250–270 nm) is strongly absorbed by the nucleic acids of microorganisms. UVC inactivates microorganisms by interrupting deoxyribonucleic acid or ribonucleic acid replication through the formation of pyrimidine dimers^10^.

Although the germicidal effect of UVC radiation was documented over many decades, the usage of UVC have been limited since overexposure to UVC radiation can potentially cause adverse effects on human health, including corneal irritation, conjunctival irritation, and skin irritation^11^. Therefore, the UVGI system has mainly been used in healthcare settings and research. Recently, the demand for household UVC germicidal devices has been increasing in response to the current COVID-19 pandemic. Commercially available UVC devices could be categorized into 2 main types based on type of UVC light source which are low-pressure mercury lamp (LPML) and light-emitting diode (LED). A wide range of applications were developed and introduced to be favorable for household-use purpose^12,13^. To the best of our knowledge, no study has evaluated the effectiveness of commercial household UVC devices for SARS-CoV-2 inactivation. Therefore, the present study aimed to measure the UVC dosage of available commercial UVC devices and to determine the effectiveness of those devices for SARS-CoV-2 inactivation.

## Materials and methods

### Study design

A prospective experimental study was conducted from February to March 2021. The study protocol was reviewed by the Institutional Review Board at Mahidol University and was considered a value-adding study (COA. MURA2021/82).

### Commercial ultraviolet C devices

**Figure 1:**
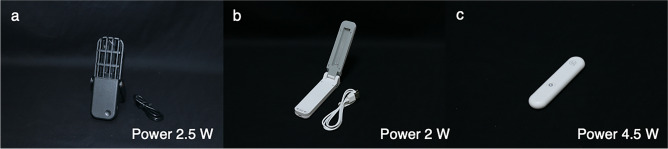
Three studied handheld ultraviolet germicidal irradiation surface disinfection equipment. All studied ultraviolet germicidal irradiation surface disinfection equipment were demonstrated (**a**; LPML1, **b**; LPML2, and **c**; LED1).

**Figure 2:**
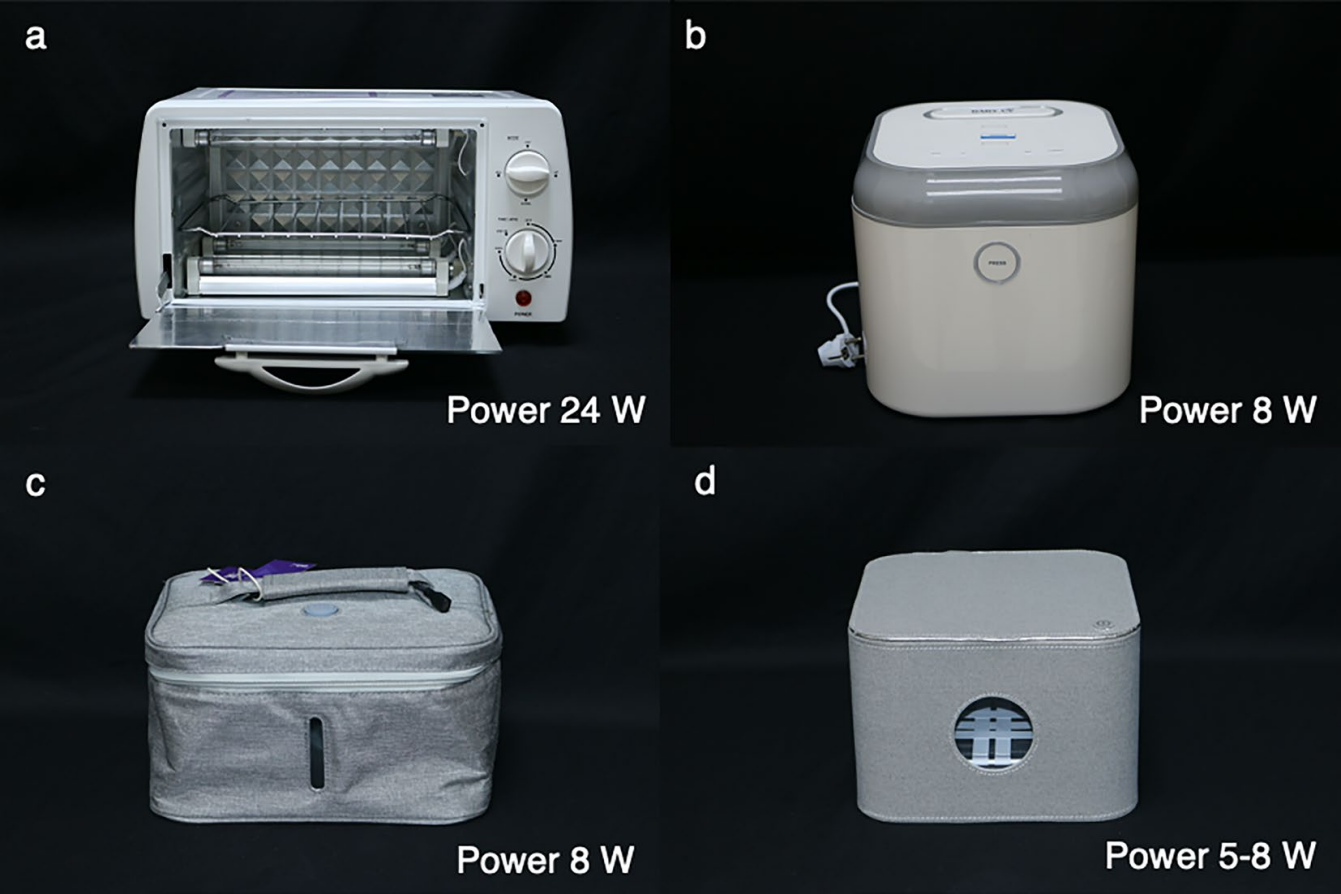
Four studied ultraviolet germicidal irradiation disinfection chambers. All studied ultraviolet germicidal irradiation disinfection chambers were demonstrated (**a**; LPML3, **b**; LPML4, and **c**; LED2, **d**; LED3).

**Figure 3:**
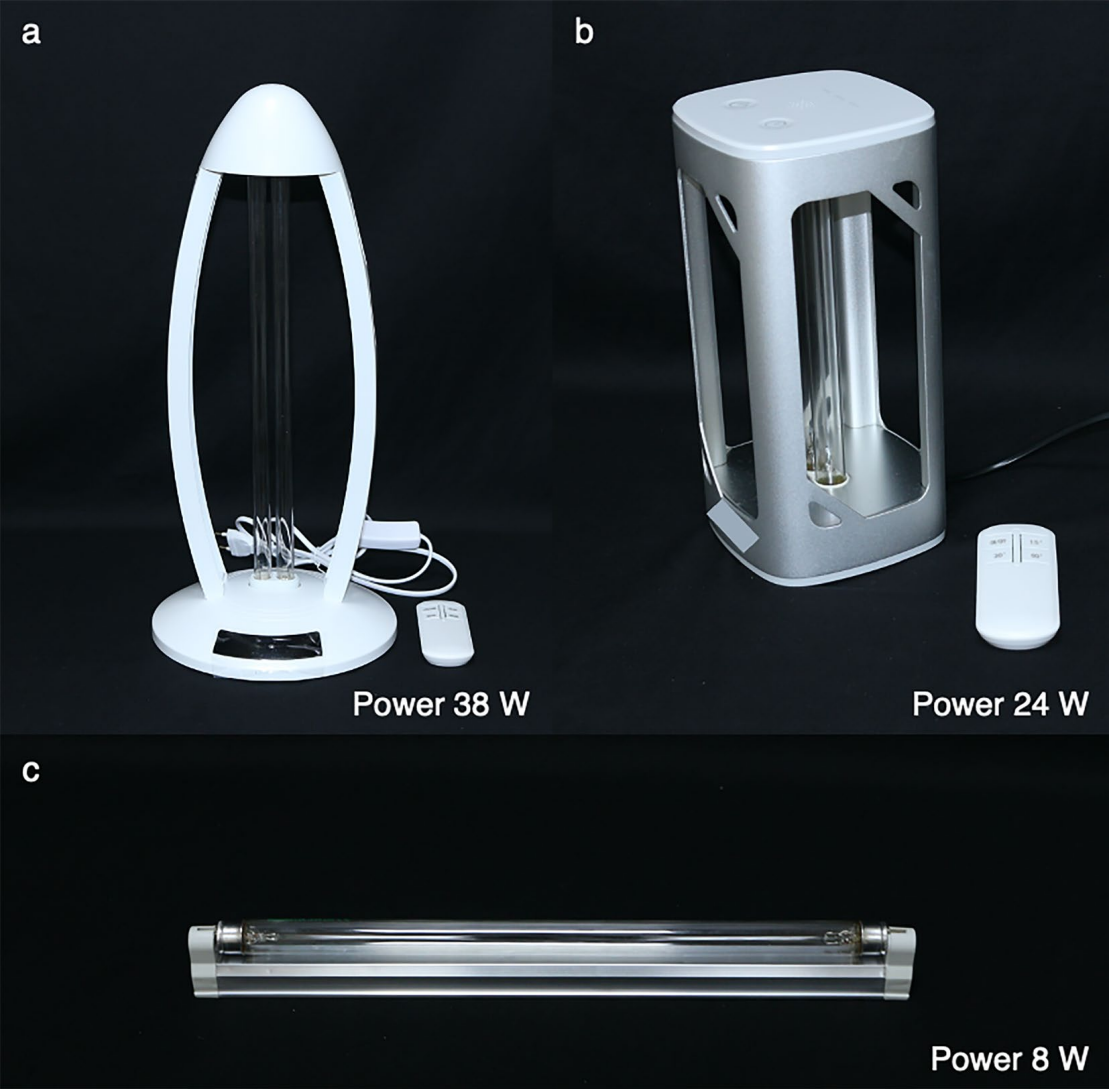
Three studied movable ultraviolet germicidal irradiation air and surface purifiers. All studied ultraviolet germicidal irradiation air and surface purifiers (**a**; LPML5, **b**; LPML6, and **c**; LPML7).

Ten UVC devices were included in the study. The devices had a household-use purpose and availability in the market. The studied devices were categorized based on UVC light source and application. Types of UVC light source were divided into LPMLs and LEDs. Applications were divided into handheld UVGI surface disinfection equipment, UVGI disinfection chambers, and movable UVGI air and surface purifiers. Regarding confidentiality, labels were assigned to each device and were used throughout the study which were described as LMPL1-7, and LED1-3 following type of UVC light source. The label and specification of UVC devices are provided in Table 1. Figures 1, 2 and 3 demonstrate all studied UVC devices classified by application and include UV light power.

**Table 1:** The label and specification of 10 studied ultraviolet C devices.

Label	UVC light source	Application	Shape Length × Width × Height (mm)	Shape of UVC lamp	UVC emission spectra (nm)	UV light power (W)	Figure
LPML1	Low-pressure mercury lamp	Handheld UVGI surface disinfection equipment	156 × 58 × 24	Compact UVC lamp	N/A	2.5	1a
LPML2			125 × 35 × 28	Compact UVC lamp	253.7	2	1b
LPML3		UVGI disinfection chamber	350 × 220 × 200	Double-ended UVC lamp (T6)	253.7	24	2a
LPML4			362 × 358 × 358	Double-ended UVC lamp (T5)	253.7	8	2b
LPML5		Movable UVGI air and surface purifier	210 × 110 × 460	Compact UVC lamp	N/A	38	3a
LPML6			120 × 120 × 247	Compact UVC lamp (2 × T16)	253.7	24	3b
LPML7			310 × 23 × 25	Double-ended UVC lamp (T5)	253.7	8	3c
LED1	Light-emitting diode	Handheld UVGI surface disinfection equipment	90 × 21 × 12	N/A	265–280	4.5	1c
LED2		UVGI disinfection chamber	260 × 140 × 190	N/A	265	8	2c
LED3			225 × 225 × 153	N/A	270–285	5–8	2d

UV, ultraviolet; UVC, ultraviolet C; UVGI, ultraviolet germicidal irradiation; N/A, not applicable.

### UVC light measurement device

**Figure 4:**
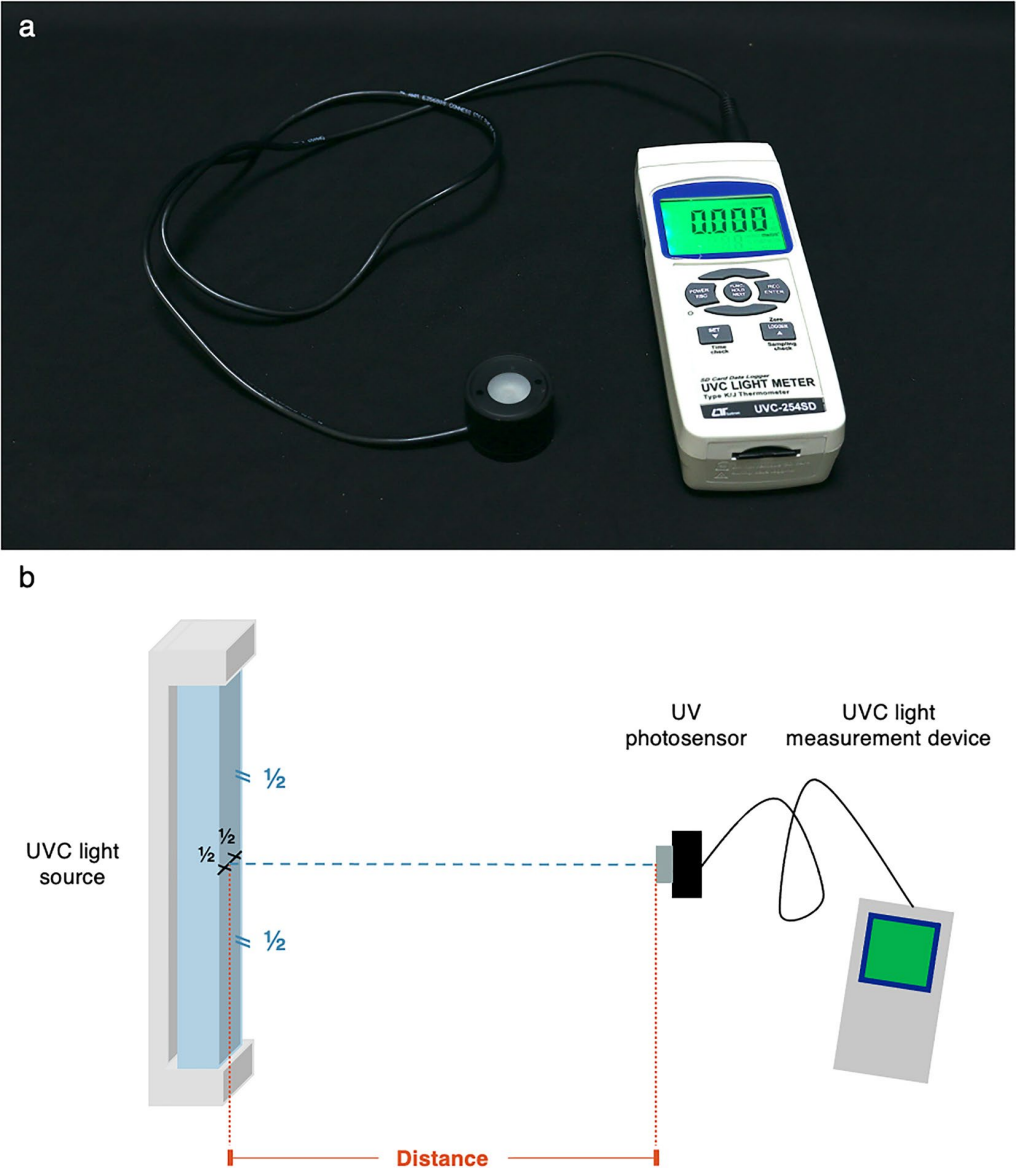
Ultraviolet light measurement device and experimental setting. A Lutron UVC-254SD meter equipped with a cosine correction filter UV photosensor was used to measure UVC irradiance (**a**). The UV photosensor was pointed directly toward UVC light source (90° angle relative to UVC light pathway) and measured at the approximate center of the UVC light source (**b**). UV; ultraviolet, UVC; ultraviolet C.

A Lutron UVC-254SD meter equipped with a cosine correction filter UV photosensor was used to measure UVC irradiance (mW/cm^2^). The sensor covered 220 nm to 280 nm UVC wavelengths (Fig. 4a).

### Experimental procedures

A Lutron UVC-254SD meter measures UVC irradiance every second. For the experimental setting, the UV photosensor was pointed directly toward the UVC light source (90° angle relative to UVC light pathway) and measured at the approximate center of the UVC light source, as shown in Fig. 4b. Duration and measurement position was different among each UVC device, determined following the manufacturers’ usage instructions. The measurement process was repeated three times under each condition.

Regarding the measured position, to evaluate the effectiveness of UVC devices in practice, UVC irradiance was categorized into short and long distances. For a short distance, we placed the sensor at a distance of 2 cm for all devices. The long distances for each device varied from 3 to 200 cm depending on the manufacturers’ usage instructions.

### Data analysis

Data of UVC irradiance was analyzed to evaluate the relationship between UVC irradiance (mW/cm^2^) and distance (cm), the relationship between UVC irradiance (mW/cm^2^) and time (sec), and SARS-CoV-2 inactivation performance.

SARS-CoV-2 inactivation performance was determined by SARS-CoV-2-inactivating UVC dosage achievement at the given duration and distance. The UVC dosage (mJ/cm^2^) is the summation of all UVC irradiance (mW/cm^2^) values obtained every second during a cycle. According to previous studies, SARS-CoV-2-inactivating UVC dosage for LPMLs and LEDs are difference. For low-pressure mercury lamps (LMPL1-7), a UVC dosage of 3.7 mJ/cm^2^ was applied as a benchmark for SARS-CoV-2-inactivating UVC dosage, which are a minimum required dose for a 3-log SARS-CoV-2 inactivation, indicating a decrease of 99.9% of the viral titer^14^. Since wavelength of UV-LED was recently founded to affect the effectiveness of SARS-CoV-2 inactivation, UVC dosage of 7 mJ/cm^2^ and 13 mJ/ cm^2^ were applied as a benchmark for LED1-2 and LED3, respectively^15^. Practical applications of each device including proper distance and time for achieving a SARS-CoV-2-inactivating UVC dosage for the studied devices were recommended.

## Results

Ten commercial household UVC devices were included. Of the 10 devices, 7 devices were LPMLs, while the others were LEDs. Data regarding shape parameters of device (mm), shape parameters of UVC lamp, UVC emission spectra (nm), and UV light power (W) were described in Table 1. No data about luminous efficiencies and semiconductor materials for LEDs was given from manufacturer. Regarding application, there were 3 handheld UVGI surface disinfection equipment (Fig. 1), 4 UVGI disinfection chambers (Fig. 2), and 3 movable UVGI air and surface purifiers (Fig. 3).

### The relationship between ultraviolet C irradiance and distance

**Figure 5:**
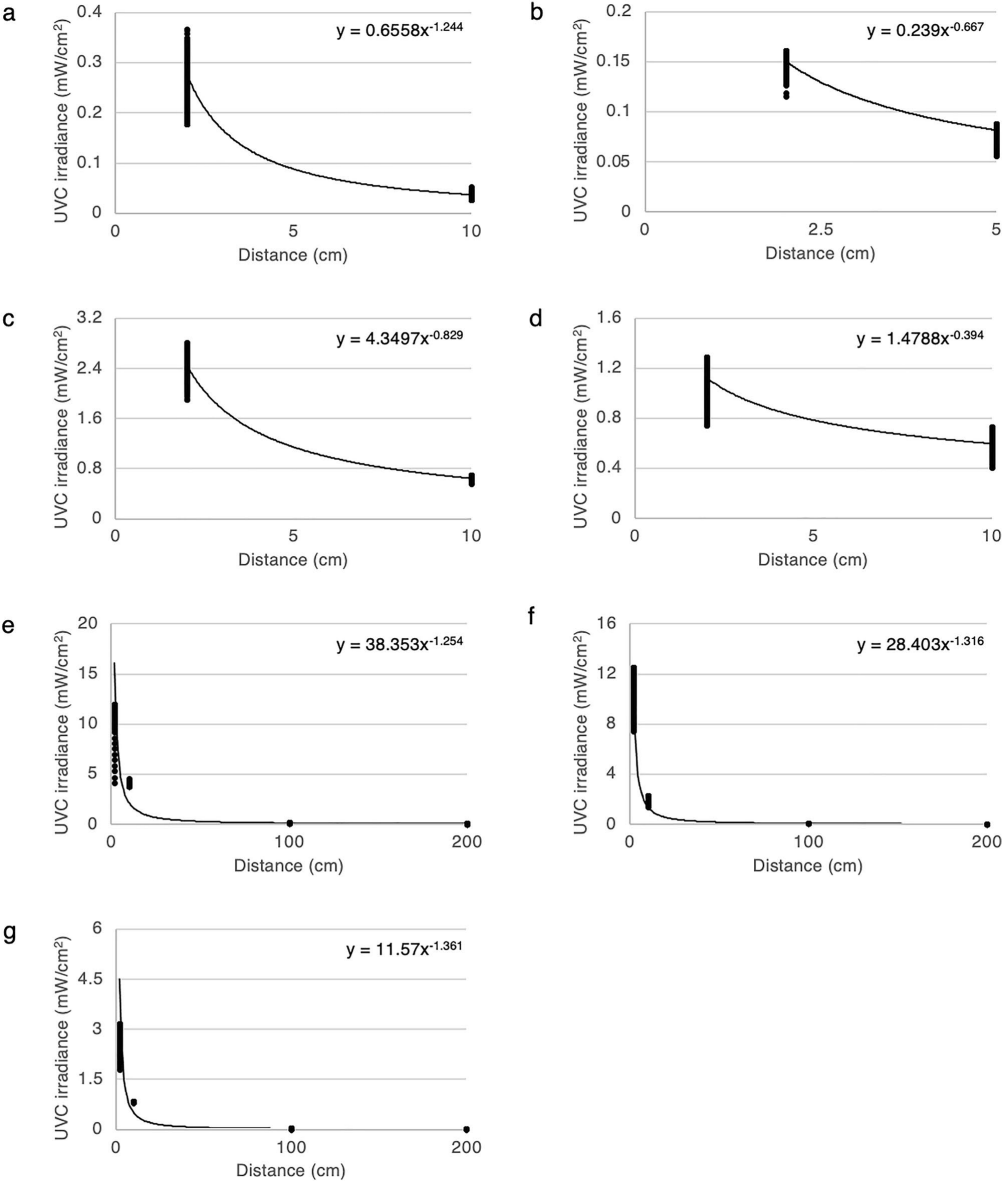
The relationship between ultraviolet C irradiance and distance of low-pressure mercury lamps. A plot of UVC irradiance (mW/cm^2^) against distance (cm) for 7 low-pressure mercury lamps (**a**; LPML1, **b**; LPML2, **c**; LPML3, **d**; LPML4, **e**; LPML5, **f**; LPML6, and **g**; LPML7) UVC; ultraviolet C.

**Figure 6:**
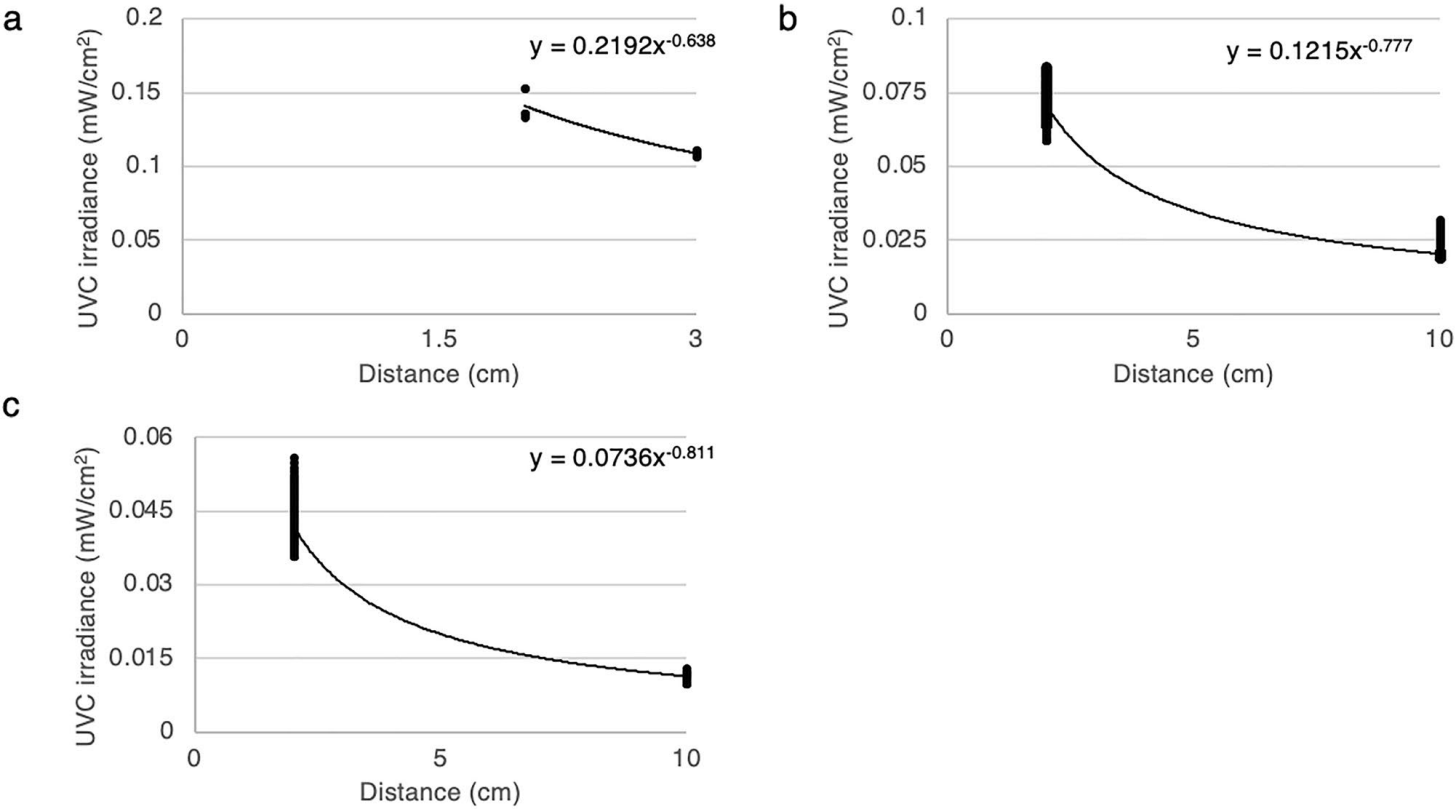
The relationship between ultraviolet C irradiance and distance of ultraviolet C light emitting diodes. A plot of UVC irradiance (mW/cm^2^) against distance (cm) for 3 ultraviolet C light emitting diodes (**a**; LED1, **b**; LED2, and **c**; LED3) UVC; ultraviolet C.

To illustrate the relationship between UVC irradiance and distance, a graph was established by plotting UVC irradiance (y-axis; mW/cm^2^) with respect to distance (x-axis; cm). Figures 5 and 6 show the relationship between UVC irradiance and distance for LPMLs (LPML1-7) and UVC-LEDs (LED1-3), respectively. The graphs showed that the UVC irradiance was inversely proportional to the distance. Different degrees of inversely proportions were founded across different UVC devices.

### The relationship between ultraviolet C irradiance and time

The plot in Supplementary Fig. S1-3 demonstrate the relationship between UVC irradiance (mW/cm^2^) and time (sec) for LPML1-4, LPML 5-7, and LED 1-3, respectively.

### Severe acute respiratory syndrome coronavirus 2 inactivation performance

For standardization and practical purposes, UVC dosages were compared across devices with similar applications. UVC dosages at short and long distance with recommended settings are shown in Table 2.

**Table 2:** Ultraviolet C dosage and recommended settings of 10 studied ultraviolet C devices.

Label	Benchmark dosage* (mJ/cm^2^)	Short distance				Long distance			
		Distance (cm)	Duration (sec)	UVC dosage (mJ/cm^2^)	Recommendation distance and duration	Distance (cm)	Duration (sec)	UVC dosage (mJ/cm^2^)	Recommendation distance and duration
**Handheld ultraviolet germicidal irradiation surface disinfection equipment**									
LPML1	3.7	2	900	260.60	2 cm, 13 s	140	1800	**0**	10 cm, 120 s
LPML2	3.7	2	30	4.24	2 cm, 27 s	5	30	**2.102**	5 cm, 52 s
LED1	7	2	2	**0.28**	2 cm, 49 s	3	2	**0.22**	3 cm, 65 s
**Ultraviolet germicidal irradiation disinfection chambers**									
LPML3	3.7	2	35	86.14	2 cm, 2 s	10	35	22.62	10 cm, 6 s
LPML4	3.7	2	660	790.22	2 cm, 5 s	10	1260	755.75	10 cm, 12 s
LED2	7	2	300	19.40	2 cm, 104 s	10	300	**6.802**	10 cm, 309 s
LED3	13	2	180	**7.58**	2 cm, 309 s	10	180	**2.05**	10 cm, 1141 s
**Movable ultraviolet germicidal irradiation air and surface purifiers**									
LPML5	3.7	2	900	9622.97	2 cm, 1 s	200	900	36.55	200 cm, 95 s
LPML6	3.7	2	900	9137.52	2 cm, 1 s	200	900	21.61	200 cm, 144 s
LPML7	3.7	2	900	2794.49	2 cm, 2 s	200	900	5.37	200 cm, 622 s

UVC, ultraviolet C.

*Benchmark dosage is UVC dosage for severe acute respiratory syndrome coronavirus 2 inactivation. Significant values are in bold.

There were 3 handheld UVGI surface disinfection equipment (LPML1, LPML2, and LED1). The long distances of LPML1, LPML2, and LED1 were 140 cm, 5 cm, and 3 cm, respectively, following the manufacturers’ instructions. According to the instructions, none of the studied handheld UVGI surface disinfection equipment achieved the SARS-CoV-2-inactivating UVC dosage. At 140 cm, no UVC irradiance was detected from LPML1. The maximum distance at which UVC irradiance could be detected was 10 cm. Considering the duration, 120 secs of LPML1 usage provided a sufficient UVC dosage for SARS-CoV-2 inactivation. At the recommended distances of LPML2 and LED1, UVC irradiances were detected for both devices; however, the SARS-CoV-2-inactivating UVC dosage was not achieved despite the use of the recommended duration. Recommended distance and duration for these 3 devices are shown in Table 2.

Four UVGI disinfection chambers labeled LPML3, LPML4, LED2, and LED3 were included in the experiment. Since the UVC light sources of the studied chambers were located in the lid, 2 cm and 10 cm from the light source could be defined as the top and bottom of the chambers, respectively. It was remarked that there were 2 light sources (located in the lid and at the floor) in 1 UVGI disinfection chamber (Fig. 2a). However, the sensor of UVC meter point toward the light source located at the lid. LPML3 and LPML4 showed favorable results. As shown in Table 2, these devices provided sufficient UVC dosage for SARS-CoV-2 inactivation when following the usage instructions from the manufacturers. In contrast, LED2 could not deliver a sufficient UVC dosage measured at the bottom of the chamber with the recommended duration. The LED3 did not achieve an adequate UVC dosage for both short and long distances.

## Discussion

UVC devices with different specifications were included in this study to represent the variety of UVC devices available on the market. SARS-CoV-2 inactivation performance was examined and categorized by device application to provide ease of use in practice.

To select the suitable UVC device for household-use, there are two main factors to be considered. First is the type of UVC light source. As mentioned, commercially available UVC devices could be categorized into LPML and LED^12,13^. LPML, or UVC discharge lamp, is a traditional UVC source mainly emitting at 253.7 nm for more than 90% of their total spectral power. The information of luminous efficiencies was not provided by manufacturer, however, LPML has 30% efficient at converting input power to UVC radiation in general^13^. Although LPML effectively generates a high radiation intensity, it has a major disadvantage that should not be overlooked. The main component (mercury) is known for its toxicity to humans and the environment^16,17^. As the accessibility of UVC devices expands, the public should be aware of mercury toxicity, and the safety of commercial mercury lamps must be ensured by manufacturers. Other important drawbacks include limited lifetime and turn-on time. To reach the expected output power, LPML usually requires few seconds to some minutes for preheating^12^. Regarding 7 LPMLs included in this study, their turn-on time ranged from 1 to 300 s (see Supplementary Fig. S1-2).

To address these limitations, LED with UVC spectrum emission were introduced to commercial markets. With its nontoxicity, compactness, and instant turn-on compensation, the popularity of UVC-LED has been gradually rising. However, low external quantum efficiency, aging of the packaging material, and thermal management problems are the main faults^12,13,18^. A LED is a semiconductor light source that emits a particular wavelength of light depending on semiconductor material and its structure. At the time, UVC-LED device is based on aluminum gallium nitride material with emission spectra of 200–280 nm^18^. The emission spectra of 3 studied LEDs were described in Table 1. Although luminous efficiencies were not given by manufacturers, the efficiency of UVC-LED varies from 1 to 3%, which is significantly lower compared to those of LPML^12,13^. As can be expected, no turn-time was required in 3 studied UVC-LEDs, which are evidently shown in Supplementary Fig. S3. Nevertheless, neither studied UVC-LED was able to produce sufficient UVC dosage for SARS-CoV-2-inactivation under manufacturers’ usage instructions, which pointed out that low external quantum efficiency is an urgent technical issue for UVC-LED needed to improve.

The second factor in selecting UVC devices is its application. A wide range of applications are commercially available but could be divided into 3 main applications: handheld UVGI surface disinfection equipment, UVGI disinfection chambers, and movable UVGI air and surface purifiers. Different applications of UVC devices possess certain characteristics that make them useful for different purposes. Specifically, handheld UVGI surface disinfection equipment is characterized as a small portable UVC device providing a minimum sufficient level of UVC irradiance for SARS-CoV-2 inactivation. Therefore, this application is appropriate for disinfecting small surface areas, such as cell phones, keyboards, and door handles. The effective range is a short distance of less than 10 cm. A practical misleading point for this application is the overestimation of the application range, as evidently seen in the LPML1 device. A longer duration of UVC irradiance could not compensate for a longer effective range.

UVGI disinfection chambers are another common UVC application suitable for surface disinfection. Importantly, the values of UVC irradiance at the top and bottom of the chamber were different. The UVC dosage at the bottom of the chamber in 2 devices (LPML3 and LPML4) was more than the dosage required for SARS-CoV-2 inactivation; therefore, the recommended duration could be shortened. However, the design of the chambers can prevent potential UVC side effects in humans; thus, the authors support manufacturers’ usage instructions to gain the benefit of inactivation of bacteria and other viruses without increasing the risk of side effects^19^.

Comparing the 3 studied applications, the UVC dosages of the movable UVGI air and surface purifiers at the recommended settings were the highest, which can be explained by their UVC light sources and usage purpose. These applications are effective not only for surface disinfection but also proper for air disinfection. Unlike UVGI disinfection chambers, UVC irradiance from movable UVGI air and surface purifiers disperses, so potential side effects to humans should be taken into consideration. Accordingly, the authors suggest a shorter irradiance duration for movable UVGI air and surface purifiers in SARS-CoV-2 inactivation and a protection for eye and skin while using these devices.

In addition to distance and duration, the direction of UVC irradiance is another crucial factor determining the disinfectant property of UVC. Boyce et al.^20^ conducted an experimental study to evaluate the impact of room location on UVC irradiance and UVC dosage. The results revealed that the orientation of the UVC sensor relative to the UVC device affected UVC irradiance. The UVC sensor pointed directly at UVC light yielded the highest UVC irradiance.

The authors are aware of the limitations of this study. First, a benchmark for the SARS-CoV-2-inactivating UVC dosage was used instead of examining SARS-CoV-2 inactivation with UVC. The second limitation is the generalizability of the results. The specifications of the studied UVC devices varied in the type of light source, application, and usage (distance and cycle duration), which could be inferred from the variety of UVC devices available in the commercial market. Consequently, the results from the present study will help guide the effectiveness of commercial household UVC devices for SARS-CoV-2 inactivation, but further adjustments are necessary depending on the specifications of the UVC device.

## Conclusion

All movable UVGI air and surface purifiers and UVGI disinfection chambers with low pressure mercury lamps emitted an adequate UVC dosage for SARS-CoV-2 inactivation, but handheld UVGI surface disinfection equipment provided a minimum sufficient level of UVC irradiance for SARS-CoV-2 inactivation. There was no standardization of the distance and cycle duration for each UVC application in achieving SARS-CoV-2 inactivation in the present study. Standard usage guidelines for UVC devices are required to improve the effectiveness of UVC irradiance for SARS-CoV-2 inactivation as well as to minimize the potential side effects of UVC.

## Supplementary Information

Supplementary Information.
