# Supplementary material for: The effectiveness of commercial household ultraviolet C germicidal devices in Thailand
Source: Sci Rep. 2021 Dec 13;11:23859. doi: 10.1038/s41598-021-03326-4 (PMC8668883; doi:10.1038/s41598-021-03326-4)
Supplement: Supplementary file 1 — Supplementary Information. [file 41598_2021_3326_MOESM1_ESM.pdf]

Title of the manuscript

The effectiveness of commercial household ultraviolet C germicidal devices in Thailand

The name(s) of the author(s)

Pasita Palakornkitti[1], MD.

Prinpat Pinyowiwat[1], MD.

Somsak Tanrattanakorn[1], MD., Asst. Prof.

Natta Rajatanavin[1], MD., Clinic Prof.

*Ploysyne Rattanakaemakorn[1], MD., Assoc. Prof.

The affiliation(s) and address(es) of the author(s)

[1]Division of Dermatology, Department of Medicine, Faculty of Medicine Ramathibodi Hospital, Mahidol University, Bangkok, Thailand

Supplementary Figure S1: The relationship between ultraviolet C irradiance and time of low-pressure mercury lamps (LPML1-4)

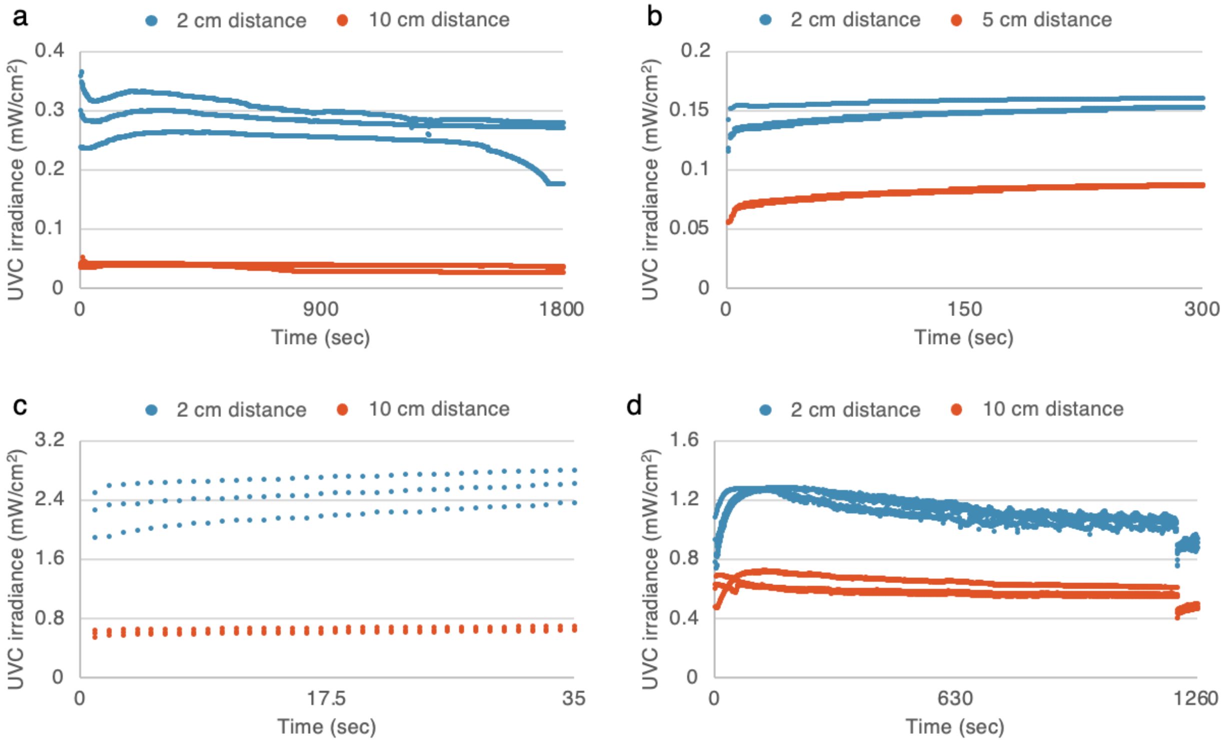

A plot of UVC irradiance (mW/cm^2) against time (sec) for 4 low-pressure mercury lamps at distances of 2 and 10 cm (a; LPML1, b; LPML2, c; LPML3, and d; LPML4)

UVC; ultraviolet C

Supplementary Figure S2: The relationship between ultraviolet C irradiance and time of low-pressure mercury lamps (LPML5-7)

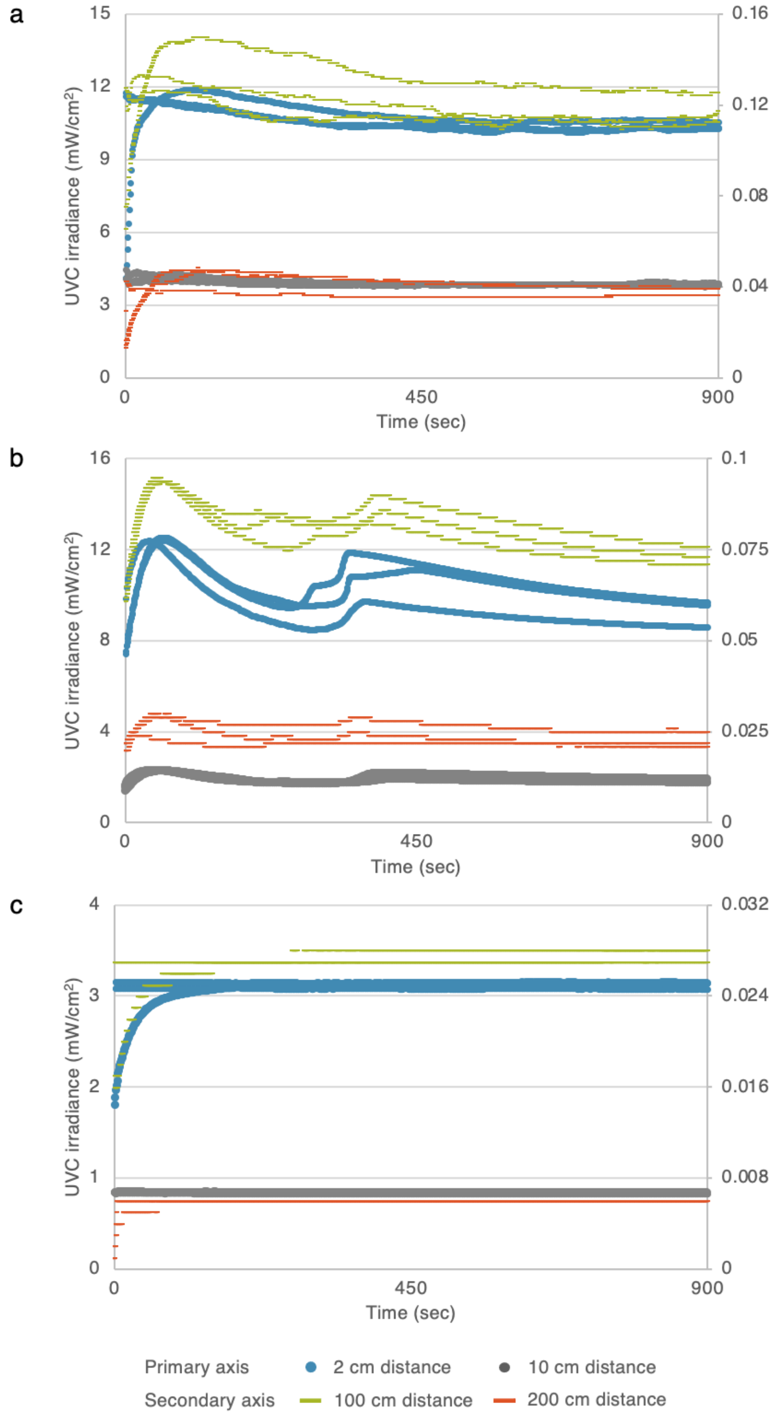

A plot of UVC irradiance (mW/cm^2) against time (sec) for 3 low-pressure mercury lamps at distances of 2, 10, 100, and 200 cm (a; LPML5, b; LPML6, and c; LPML7)

UVC; ultraviolet C

Supplementary Figure S3: The relationship between ultraviolet C irradiance and time of ultraviolet C light emitting diodes (LED1-3)

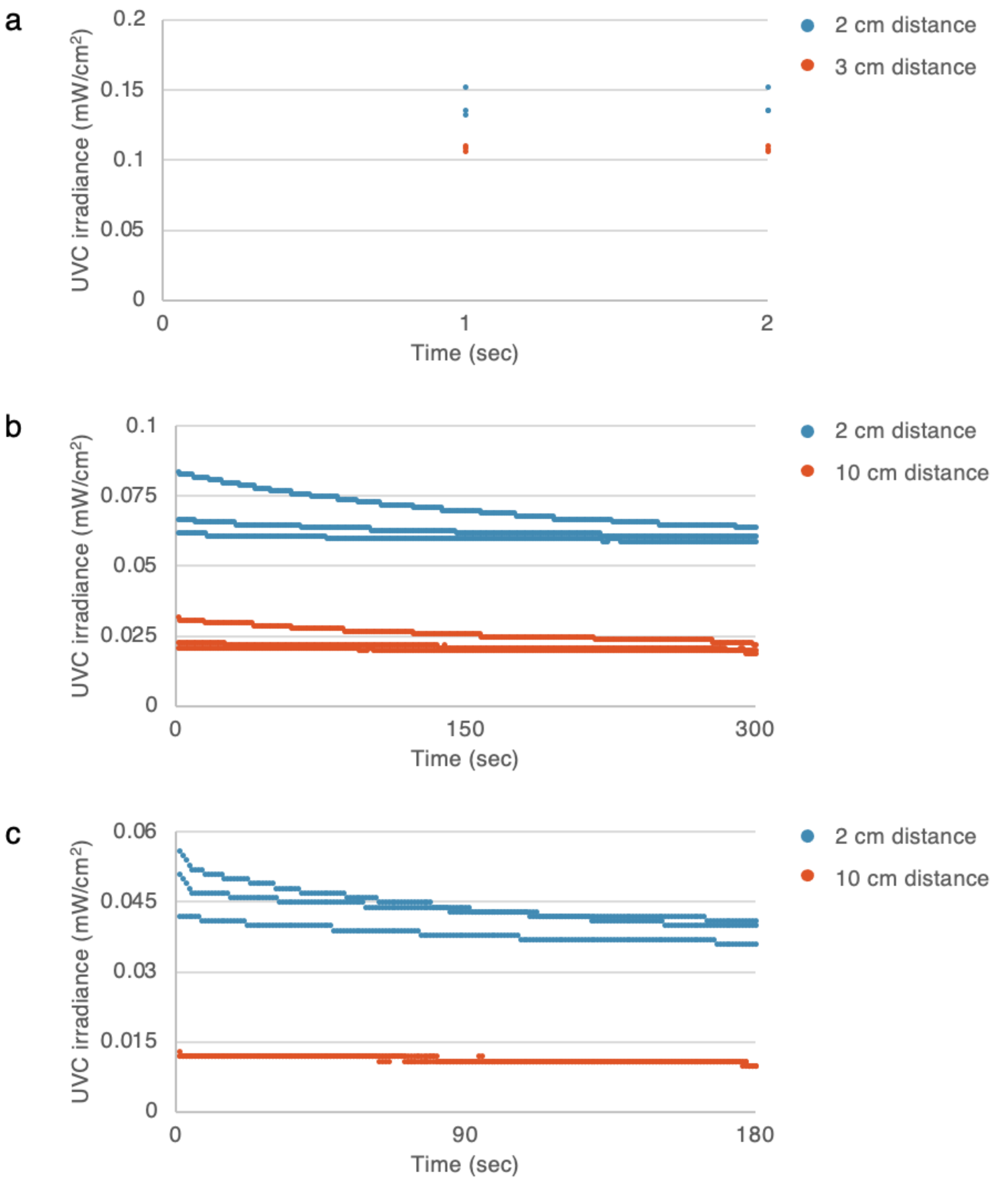

A plot of UVC irradiance (mW/cm^2) against time (sec) for 3 ultraviolet C light emitting diodes at distances of 2 and 10 cm (a; LED1, b; LED2, and c; LED3)

UVC; ultraviolet C
